# Intraperitoneal Glucose Transport to Micrometastasis: A Multimodal In Vivo Imaging Investigation in a Mouse Lymphoma Model

**DOI:** 10.3390/ijms22094431

**Published:** 2021-04-23

**Authors:** Zsombor Ritter, Katalin Zámbó, Xinkai Jia, Dávid Szöllősi, Dániel Dezső, Hussain Alizadeh, Ildikó Horváth, Nikolett Hegedűs, David Tuch, Kunal Vyas, Péter Balogh, Domokos Máthé, Erzsébet Schmidt

**Affiliations:** 1 Department of Medical Imaging, University of Pécs Medical School, 7624 Pécs, Hungary; ritterzsombor@gmail.com (Z.R.); zambo.katalin@pte.hu (K.Z.); dezso.daniel@pte.hu (D.D.); 2 Department of Immunology and Biotechnology, University of Pécs Medical School, 7624 Pécs, Hungary; jia.xinkai@pte.hu (X.J.); balogh.peter@pte.hu (P.B.); 3 Department of Biophysics and Radiation Biology, Faculty of Medicine, Semmelweis University, 1094 Budapest, Hungary; szollosi.sote@gmail.com (D.S.); panpills@gmail.com (I.H.); hegedus.nikolett@med.semmelweis-univ.hu (N.H.); 4 1st Department of Internal Medicine, University of Pécs Medical School, 7624 Pécs, Hungary; alizadeh.hussain@pte.hu; 5 CROmed Ltd., 1047 Budapest, Hungary; 6 Lightpoint Medical Ltd., Chesham HP51PE, UK; david.tuch@lightpointmedical.com (D.T.); kunal.vyas@lightpointmedical.com (K.V.); 7 Hungarian Centre of Excellence for Molecular Medicine, In Vivo Imaging ACF, 1094 Budapest, Hungary

**Keywords:** Cerenkov light imaging, image-guided surgery, lymphoma, peritoneal metastasis, molecular imaging, micrometastasis, PET/MRI, fiberoptic microscopy, FDG

## Abstract

Bc-DLFL.1 is a novel spontaneous, high-grade transplantable mouse B-cell lymphoma model for selective serosal propagation. These cells attach to the omentum and mesentery and show dissemination in mesenteric lymph nodes. We aimed to investigate its early stage spread at one day post-intraperitoneal inoculation of lymphoma cells (n = 18 mice), and its advanced stage at seven days post-inoculation with in vivo [18F]FDG-PET and [18F]PET/MRI, and ex vivo by autoradiography and Cherenkov luminescence imaging (CLI). Of the early stage group, nine animals received intraperitoneal injections, and nine received intravenous [18F]FDG injections. The advanced stage group (n = 3) received intravenous FDG injections. In the early stage, using autoradiography we observed a marked accumulation in the mesentery after intraperitoneal FDG injection. Using other imaging methods and autoradiography, following the intravenous injection of FDG no accumulations were detected. At the advanced stage, tracer accumulation was clearly detected in mesenteric lymph nodes and in the peritoneum after intravenous administration using PET. We confirmed the results with immunohistochemistry. Our results in this model highlight the importance of local FDG administration during diagnostic imaging to precisely assess early peritoneal manifestations of other malignancies (colon, stomach, ovary). These findings also support the importance of applying topical therapies, in addition to systemic treatments in peritoneal cancer spread.

## 1. Introduction

The 2-deoxy-2-[18F]fluoro-D-glucose ([18F]FDG) molecule is the most commonly used radiotracer in clinical nuclear medicine for diagnostic imaging, and is mostly transported to the cells via upregulated glucose transporter proteins (GLUT1-7) [1,2]. In recent years, [18F]FDG-positron emission tomography/X-ray computed tomography (PET/CT) has become the key component of diagnostic assessment and is used to determine the staging of patients with high-grade lymphoma, particularly diffuse large B-cell lymphoma (DLBCL) and Hodgkin’s disease (HD) [3,4]. According to clinical experience, patients with lymphoma often have abdominal manifestations [5]. In addition to staging, FDG is used to assess responses to therapy, and the most recent publications have also shown the prognostic potential of FDG-PET/CT [6,7,8,9,10].

Bc.DLFL1 is a spontaneous high-grade B-cell lymphoma isolated from a BALB/c mouse, which is reproducibly transplantable via intraperitoneal injection into syngeneic recipients. After intraperitoneal inoculation, the lymphoma cells attach to lymphocyte-rich regions within the omentum and mesentery, and subsequently disseminate towards the mesenteric lymph nodes [11]. Bc.DLFL1 is therefore a suitable model for preclinical investigations of the peritoneal spread of DLBCL [12].

In the present work, we aimed to investigate the early spread of Bc.DLFL1 lymphoma in vivo with [18F]FDG-PET and positron emission tomography/magnetic resonance imaging (PET/MRI), and ex vivo by autoradiography and Cherenkov luminescence imaging (CLI) using [18F]FDG. The results of these imaging techniques were correlated with immunohistochemical tissue analysis.

We hypothesized that in the case of early tumor spreading in the omentum and mesenterium, cell groups lacking significant vascularization would not be detected with an intravenously administered radiopharmaceutical. This raises the potential need for topical radiopharmaceutical administration in addition to intravenous diagnostic procedures and therapeutic approaches. Therefore, we tested our diagnostic approach using multiple modalities of imaging in the mouse Bc.DLFL1 lymphoma model. Our findings indicate that there is a significant discrepancy in detectability via intravenous or topical radiotracer application for the early monitoring of tumor progression.

## 2. Results

### 2.1. Monitoring of Early Distribution of Bc.DLFL1 Lymphoma Cells after Intraperitoneal Inoculation Using Systemic and Topical [18F]FDG Administration

The typical radiotracer-based approach for in situ tumor detection employs the preferential uptake and cellular entrapment of [18F]FDG molecules by tumor cells. To assess whether Bc.DLFL1 cells accumulate [18F]FDG, first we incubated freshly isolated Bc.DLFL1 lymphoma cells in vitro in the presence of 100 kBq radioactivity of [18F]FDG, and compared their tracer uptake to that of normal mouse leukocytes in triplicate samples. The average [18F]FDG uptake was 48% of the original input activity by lymphoma cells, and 3% of the original activity by normal mouse mixed leukocytes. These observations indicate that Bc.DLFL1 lymphoma cells have a significantly higher capacity to take up [18F]FDG over normal mouse white blood cells; thus, [18F]FDG appears to be a sensitive in vivo tracing tool for this lymphoma (Table 1).

**Figure 1:**
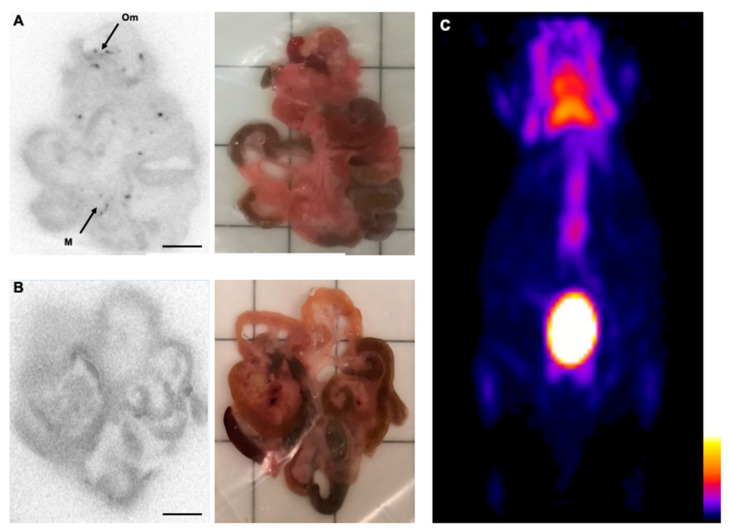
Intraperitoneal FDG is superior for the detection of intravenously administered molecules in the early stage Bc.DLFL1 mouse lymphoma model. Ex vivo autoradiographic detection of the tumor cells 24 h post-intraperitoneal inoculation. The FDG injections revealed that cells adhered to the omentum (Om) and mesentery (M) placed on the ARG cassette (**A**). Scale bar represents 1 cm in the images. Intravenous administration of the radiotracer failed to identify mesentery- and omentum-associated lymphoma cells (**B**). The corresponding white light image panel is also presented for orientation purposes. The lack of a tumor-derived signal 24 h after lymphoma inoculation is shown with a PET three dimensional maximal intensity projection reconstruction acquired 2 h after intraperitoneal FDG application (**C**). Only normal FDG mouse distribution is visible in the PET image, its color scale representing radioactive concentrations in kBq/mL.

To determine whether in vivo PET imaging methods would allow monitoring of disease progression at the early stage, we used [18F]FDG for high grade lymphoma detection. We injected the Bc.DLFL1 lymphoma cells intraperitoneally, which was followed by the administration of the radiopharmaceutical 24 h later via intraperitoneal or intravenous injection. Using a whole body PET scan, we could not detect specific tracer accumulation in the early stage (1 day post-inoculation) after either intraperitoneal or intravenous tracer administration. To ensure that the tracer reached the potential omental and mesenteric propagation sites, we performed ex vivo autoradiography of the entire intestinal tract. We found that 2 h after intraperitoneal FDG administration, several clearly marked foci were present within the mesentery or omentum in mice injected with Bc.DLFL1 cells; however, when scanning after the intravenous injection of FDG, no such pattern was detectable (Figure 1).

**Figure 2:**
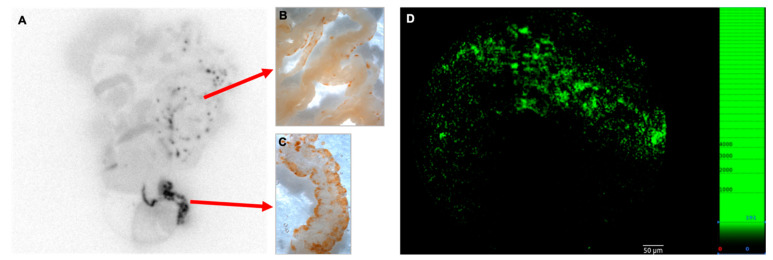
Multimodal correlated images of preferential sites during early lymphoma spreading. Correlation of ex vivo autoradiographic image of the abdominal organs (**A**) in a mouse 24 h after intraperitoneal inoculation of 5 × 10^6^ tumor cells (pre-loaded with CFSE dye) with anti-FITC immunodetection (visualized as brown DAB precipitate) of lymphoma cells, which demonstrates a matching pattern in the mesentery (M) and the omentum (Om; **B**,**C**). The diagnostic radiotracer ^18^F-FDG molecule was injected intraperitoneally 2 h before imaging (n = 9). Direct fluorescence visualization of fluorescein ex vivo using fluorescence confocal fiberoptic microscopy of the omentum revealed small- to medium-sized cell clusters (**D**).

To confirm that the selective focal omental and mesenteric ex vivo [18F]FDG signal is due to the previous local accumulation of lymphoma cells, we intraperitoneally injected BcDLFL.1 cells labeled with carboxyfluorescein (CFSE), and their distribution was monitored by immunohistochemical detection with an anti-FITC antibody. Using this technique, we found a labeling pattern matching the distribution observed by autoradiography in the omentum and the perivascular adipose cuffs surrounding the mesenteric arteries, as reported earlier [11,12]. Thus, our in situ autoradiography results following intraperitoneal administration of [18F]FDG support the immunohistochemical data on the non-random early distribution of lymphoma cells (Figure 2A–C). As a further confirmation of selective in vivo binding of CFSE-labeled lymphoma cells to the serosal lining, we used ex vivo confocal fiber-optic fluorescent microscopy (Figure 2D). We found that within the mesentery, the CFSE-marked lymphoma cells showed a focal accumulation in a pattern similar to that observed with anti-FITC immunohistochemistry. Taken together, these findings indicate that although Bc.DLFL1 lymphoma cells clearly adhere to select peritoneal locations, their [18F]FDG tracing after either intravenous or intraperitoneal isotope administration does not permit in vivo lymphoma detection, while ex vivo [18F]FDG monitoring can reveal lymphoma accumulation after intraperitoneal tracer administration.

### 2.2. Successful Monitoring of Lymphoma Expansion in Nodal Metastasis by PET/MR and Subsequent CLI

**Figure 3:**
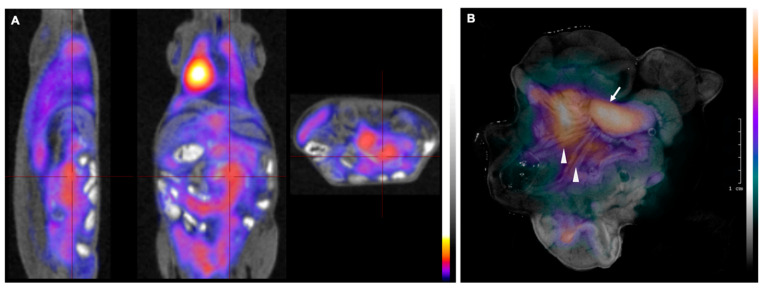
PET/MRI images of Bc-DLFL.1 lymphoma cell biodistribution in the advanced stage. PET/MRI images of Bc.DLFL1 cell biodistribution at advanced lymphoma stage after intraperitoneal tumor inoculation detected by intravenous FDG injection (administered 1 h earlier) show several mesenteric lymph nodes accumulating (**A**). After subsequent removal, the abdominal organs were subjected to CLI ex vivo. Panel B shows superimposed white and Cerenkov light pictures. The arrow indicates extensive FDG accumulation in the mesenteric lymph node conglomerates (arrow). In addition to mesenteric lymph nodes, CLI also detected the lymphoma expansion within mesenterial fatty streaks (arrowheads) (**B**).

**Figure 4:**
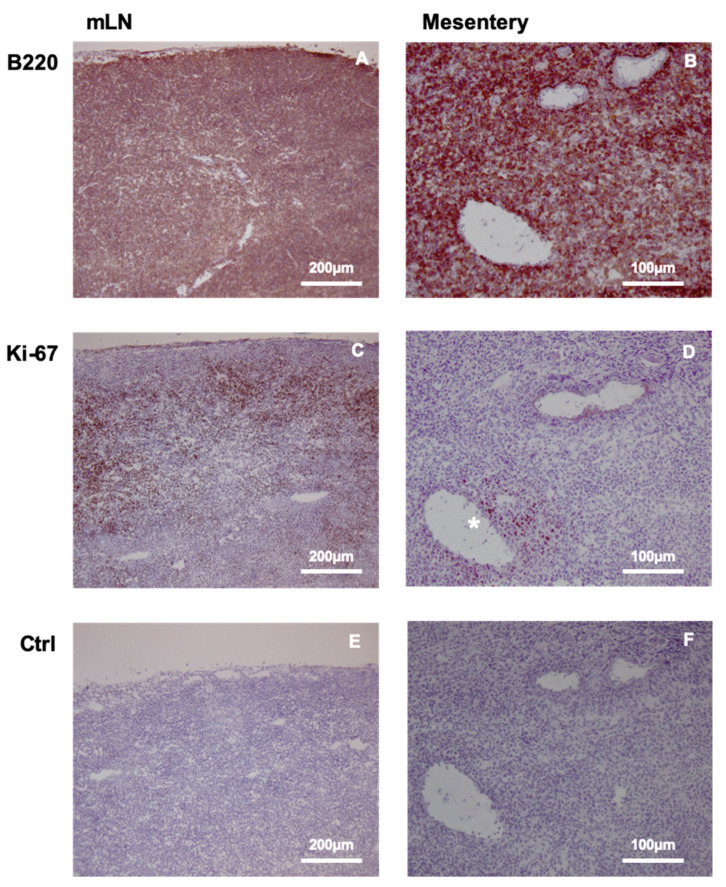
DLBCL accumulation and proliferation in mesenteric lymph node (mLN) and mesentery. B220 staining of mLN (**A**) demonstrates lymphoma expansion throughout the tissue, while the expression of proliferation marker Ki-67 (**C**) reveals preferential proliferation in the cortical region, and less prominent division is observed in the medullary area. The mesentery also contains diffusely distributed B220-positive DLBCL cells (**B**), with dividing cells positive for Ki-67 marker restricted to the area surrounding the mesenteric vein (**D**) (labeled with *). Negative control staining of mLN and the mesentery are shown in (**E**,**F**).

Our previous findings indicated the preferential expansion of Bc.DLFL1 lymphoma cells after intraperitoneal injection in the mesenteric lymph nodes (mLNs) and spleen. To monitor the expansion of tumor at the late stage after the initial adhesion to serosal foci, we tested whether PET or CLI would allow lymphoma detection 7 days after inoculation. We found that, in contrast to the lack of radiopharmaceutical signal at the early lymphoma stage, a robust accumulation of [18F] FDG was detectable following the intravenous tracer administration. The most intense labeling was observed using PET/MRI in the enlarged mLNs (Figure 3A). On the other hand, this approach did not allow the separate visualization of the mesentery with the precise identification of mesenteric branches, which also harbors a substantial amount of lymphoma cells, as demonstrated by immunohistochemical analyses (Figure 4).

To improve the resolution of PET imaging of mesenteric branches, we employed Cherenkov luminescence imaging as an ex vivo imaging modality with improved resolution. Using this approach we found luminescence signal linked to [18F]FGD accumulation in the omentum and in the adipose tissue along the mesenteric vessels and lymphatics, in addition to the intense signal from the enlarged mLNs (Figure 3B). These findings reveal that at a later stage of lymphoma, the nodal metastases can be identified using PET/MRI, even after the intravenous administration of tracer, but only with marginal separation of lymph nodes and surrounding adipose tissues of the mesenterium. On the other hand, application of CLI as an ex vivo test can sufficiently identify both nodal and adipose lymphoma infiltrates.

## 3. Discussion

The present work addressed the in vivo radio-imaging possibilities for the early detection of peritoneal spreading of a high-grade B-cell lymphoma in an experimental model. Although the specific molecular components, cell types, and mechanisms that lead to lymphatic spread and peritoneal metastasis of various tumors have been extensively studied [13,14,15], the specific conditions promoting the serosal propagation of lymphoid malignancies have not been completely understood, despite their reported appearance in abdominal adipose tissues [16]. Most frequently, the tumors of the gastrointestinal tract and the ovaries may give rise to peritoneal metastases, but in the case of lymphomas, peritoneal or even mesenteric manifestations also commonly occur [16]. Therefore, our preclinical studies, performed in animal models in vivo and ex vivo, have the potential to translate diagnostic ideas and procedures to the clinical field to detect the spread of abdominal tumors. In addition, these findings may be extended to other malignancies with a more frequent occurrence of peritoneal metastases or extranodal manifestations in the abdominal cavity during the course of the disease. The detection of peritoneal metastases at an early stage could be a key component of effective therapy for these diseases [17,18].

The restricted in vivo propagation of Bc-DLFL.1 lymphoma cells in the abdomen, and in mesenteric lymph nodes at advanced stage of the lymphoma, was described previously [11]. Thus, we hypothesized that our well-characterized high-grade lymphoma isolated from BALB/c mice would be a suitable model for a preclinical investigation of various stages of abdominal tumor spread. To this end, we explored a combined molecular imaging approach, based on the widely available radiopharmaceutical [18F]FDG molecule.

The [18F]FDG molecule is the most frequently applied molecule used to diagnose high glucose transporter protein (GLUT1-7) expression of human tumor cells related to their increased glycolysis. Compared to normal tissues, tumor cells have a greater utilization of glucose: These cells generally show increased expression of GLUT transporters (mainly GLUT-1 and GLUT-3 in lymphoma), higher hexokinase activity, and decreased or absent glucose 6 phosphatase activity [19,20,21]. Therefore, we first aimed to determine [18F]FDG uptake by murine lymphoma cells, comparing results with those of normal lymphocytes. The substantially higher (around 15-fold) accumulation of [18]FDG in vitro suggested its potential for tumor imaging difference, and made it unlikely that lymphocytes would be able to generate comparable signal strength. The main role of tumor cells in FDG uptake had been already pointed out in another study examining peritoneal tumor spread in mice [22].

We tried to investigate the early distribution pattern of high-grade B-cell lymphoma (Bc.DLFL1) using a functional imaging method that can also be applied in clinical diagnostics. It is important that in addition to staging of lymphoma and many other tumors, intravenous [18F]FDG has also been applied for the PET/CT diagnosis of peritoneal carcinosis, with various reported specificities and sensitivities [23,24]. In human diagnostics, [18]FDG is always administered intravenously, and the imaging begins approximately 1–2 h after administration in subjects.

In our work, the failure to detect the serosal expansion of lymphoma by PET early after its inoculation can at least partly be attributed to the minuscule size of metastatic foci. These foci, with a size range of 0.1–0.2 mm, have a decreased contrast and are too small to work with, even using small animal PET instruments. In our early stage lymphoma monitoring, the size of tumor attached to the omental and mesenteric adipose tissue was clearly well below that of the lowest small animal PET resolution threshold of 0.7 mm [25]. In addition, while the serosal attachment sites for lymphoma binding contain a rich capillary meshwork [12], 24 h after intraperitoneal injection, only a few Bc.DLFL1 cells had entered the deeper vascularized parts of the adipose parenchyma. It seems plausible that initially, the low vascular supply due to the small size of serosal lymphocyte clusters combined with the low number of lymphoma cells that had entered the vascularized compartment, together hampered the initial identification of lymphoma adherence by intravenous FDG administration. Importantly, the vasculature in both omental and mesenteric milky spots displayed angiogenetic features [13]; thus, the subsequent invasion to these regions would probably allow for the intravenous tracing of lymphoma metastases at a later period. During the development of mouse molecular imaging techniques, the intraperitoneal administration of [18F]FDG has been proven to be a more convenient technique than intravenous injection, even though the clearance kinetics to the circulation are somewhat slower [26,27,28]. However, the detection of early intraperitoneal spread of tumors of such small sizes has not been performed in this way, even in mouse models [29,30,31]. The access of intravenously administered [18F]FDG to the early peritoneal metastatic foci is hampered by the lack of vasculature. At this early stage, the cells attached to the mesentery can proliferate, even in the absence of significant vascular supply, which can be explained by their reprogrammed metabolism [32] towards glycolysis. Although the initial adhesion territories were small, we found that the use of explant autoradiography with a better resolution potential [33,34] could efficiently resolve the location of in vivo [18F]FDG-labeled foci matching whole-mount immunohistochemistry of CFSE-marked lymphoma cells and Cellvizio confocal fiber-optic imaging. Thus, the successful identification of initial metastatic locations required intraperitoneal delivery of FDG, and its detection with a more sensitive method such as autoradiography.

In the case of larger metastatic foci, which have a size detectable with clinical PET/CT (generally if the tumor diameter reaches approximately 5–6 mm [1,2]) the problem of low vascularization might still persist; however, even clinical PET would allow the detection of these foci if an appropriate (i.e., intraperitoneal) [18F]FDG transport route were to be used. Our work points out the possibility to expand the scope of PET/CT staging, by intraperitoneal tracer injection in tumors with peritoneal spread that might remain ’silent’ if just intravenous [18F]FDG was applied. This may highlight the necessity for intraperitoneal delivery of tracer molecules in human subjects for more reliable identification of hitherto undetectable tumor colonies. This concept parallels the principles of local peritoneal administration of therapeutic compounds already in use [17,35,36,37].

After a few days, intraperitoneally-injected lymphoma cells reach the mesenteric lymph nodes where they continue to proliferate. This is confirmed by intense Ki-67 expression, and also by the substantial enlargement of lymph nodes detectable after 7 days of lymphoma inoculation. At this stage, the metastatic mLNs have become resolvable, even after intravenous FDG administration with in vivo PET. It remains to be seen whether this increased ^18^F-FDG uptake at a later stage reflects lymphoma cell expansion only, or whether inflammatory infiltration by peritoneal macrophages also partly contributes to the enhanced signal, as previously described in a peritoneal adenocarcinoma model [22]. However, we observed contradictory findings between the lymphoma-filled mesentery by immunohistological results, and its less clearly defined imaging presentation by PET in live animals. As another opportunity for radioisotope detection of FDG, we chose to use CLI, which facilitated rapid evaluation [38,39]. Positive PET scans also hinted at the presence of FDG-generated signals after the intravenous injection of tracer, due to the accumulation of lymphoma cells in vascularized mLNs, although their tissue resolution between mLNs and adjacent omental and mesenteric adipose tissues was unclear. The more precise resolution provided by the CLI approach could discriminate between the mLNs, serosal adipose tissue harboring lymphoma, and the burden-free intestinal tract. Similar to the radiodiagnostic approaches used at the early stage, at an advanced stage of lymphoma, the combined use of various imaging modalities may provide a more accurate readout of the tumor stage.

Even a decade ago, the first CLI imaging systems had a minimal detectable activity (MDA) figure of 45 kBq of FDG radioactivity in a volume of 0.3 mL for a 5 min scan time [39,40]. The LightPath^TM^ system used in the study had an MDA of 20 kBq in a scan time of 100 s and a volume of 0.02 mL. The current clinical PET technology allows for the detection of 1 MBq in 1 mL in a scan duration of 60 min (based on the authors’ own clinical PET experience). This largely corresponds to at least one magnitude of gain in sensitivity when this CLI system is applied together with a PET scan. The image resolution gain is *par excellence* two orders of magnitude for CLI.

Translating our results to clinical practice might be directly feasible. The application of CLI in ex vivo samples could follow a PET scan within hours, or even in an overnight setting. The results of that preceding PET scan could direct the surgical removal of tumorous nodal samples. Thereafter, the exact nodal sample parts to be processed during pathology could be quickly read using the ex vivo CLI scans of those removed samples. The superior resolution and signal sensitivity of CLI might give an excellent indication of where exactly the metastatic foci may reside in a sample. However, practical clinical workflow limitations should be efficiently challenged during this translation. This could involve the just-in-time organization of PET scan, PET reading, and biopsies on the same day. Excessive physical sizes of other parenchymatous tumor samples would hamper Cerenkov light transmission; therefore, those should be addressed with special CLI and tissue preparational application protocols. The main physical limitation of the widespread use of CLI-PET combinations would, however, be healthcare staff radiation exposure.

## 4. Methods

### 4.1. [18F]FDG Uptake Test of Bc.DLFL1 Mouse Lymphoma Cells In Vitro

Mesenteric lymph nodes of two lymphoma-bearing and two healthy BALB/c mice were collected and homogenized. The homogenates were filtered through 70 µm cell strainers and centrifuged. The cells were counted in a Bürker chamber and the preparation was diluted to the desired cell density. We compared the [18F]FDG uptake of 10^7^/mL lymphoma cells to that of 10^7^/mL mixed lymphocytes from healthy animals. Two comparative measurements (Hidex Triathler, Hidex, UK) were performed under the same conditions in triplicates. The mixed leukocyte cells were then subjected to the same incubation procedure as the lymphoma cells (see below). The freshly isolated cells were maintained in physiological saline for 1 h and then centrifuged (5000× *g* for 5 min) at 37 °C. At the same temperature, 1 mL of [18F]FDG solution in physiological saline with a radioactivity of 100 kBq was added to the cell pellet and was thoroughly mixed. After 1 h of incubation time for tracer uptake, the cells were centrifuged, the fluids (supernatant-1) were removed, and their activity was measured. After re-suspension and repeated centrifugation, the second set of fluids (supernatant-2) was removed and the activities of both the cell pellet and supernatant-2 were measured. The distribution ratio of [18F]FDG between the cell pellet and the sum of supernatants-1 and -2 was calculated.

### 4.2. Experimental Animals and Bc-DLFL.1 Lymphoma Propagation

BALB/c mice aged between 8–12 weeks were bred at the SPF Animal Breeding Unit and were used as lymphoma recipients. After retrieval, the mice were adopted to a conventional animal facility of the Department of Immunology and Biotechnology. The Bc.DLFL1 lymphoma cells were maintained as serial intraperitoneal passage in mice. All procedures involving live animals were carried out in accordance with the guidelines set out by the Ethics Committee on Animal Experimentation (University of Pécs, Pécs, Hungary) under license number BA02/2000-16/2015 and conformed to the ARRIVE guidelines.

A total of 5 × 10^6^/mL lymphoma cells in DMEM tissue culture medium (Sigma-Aldrich, St. Louis, MO, USA) were incubated in 5µM 5-6-carboxyfluorescein succinimidyl ester (CFSE) dissolved in DMSO at 37 °C for 20 min, as previously described [41]. After washing in DMEM containing 10% fetal bovine serum, the cells were adjusted to 10^6^ cells/recipient in 0.5 mL, and were injected intraperitoneally.

Mice were grouped into an early stage group, where imaging was performed one day post-inoculation of lymphoma cells (n = 18 mice), and an advanced stage group with imaging of n = 3 mice performed 7 days post-inoculation of the cells.

In the early stage group, nine animals received intraperitoneal injection of FDG and nine mice received FDG via the intravenous administration route in the lateral tail vein. The advanced stage group received intravenous FDG injections.

### 4.3. In Vivo Imaging Using [18F]FDG PET

Each animal received 10–15 MBq FDG (Pozitron-Scan^®^, Pozitron, Hungary) in 0.1 mL injection 1 h before the PET and 1.5 h before the subsequent CLI imaging. Animals were not fasted prior to imaging. PET were acquired in a Micropet P4 (Concorde Microsystems, Knoxville, TN, USA) small animal PET system. Static scans of 15 min duration were collected 90 min post injection in an energy window of 350–750 keV. All PET images were reconstructed using the three-dimensional Maximum A Posteriori (MAP, P4 system) algorithm, with corrections for scatter and attenuation where available. Reconstructed images of 1 mm voxel size were then visualized using the VivoQuant (inviCRO, Boston, MA, USA) and Fusion (Mediso, Budapest, Hungary) software.

### 4.4. In Vivo Imaging Using PET/MRI

Mouse PET/MRI measurements were performed in a MultiCell™ heating, positioning and monitoring multi-animal bed on an nanoScan 1T integrated imaging systems (both Mediso Ltd., Budapest, Hungary). Ninety minutes after FDG injection, a 15 min static PET data acquisition was obtained in an energy window of 350–750 keV, immediately followed by a three-dimensional T1-weighted gradient echo sequence, with 300 micron voxel size, 6 excitations, 12.1 ms/2.9 ms Transmit/Receive times, and 15 degrees of flip angle. Quantitative radioactivity PET data were reconstructed using a Monte-Carlo based iterative algorithm (Tera-Tomo™, Mediso Ltd., Budapest, Hungary) using the MRI as anatomical and attenuation priors, with 0.3 mm voxel size for a whole-body PET/MRI image with a resolution of 1 mm.

### 4.5. Processing of Ex Vivo Tissues for Autoradiography and CLI

Either 24 h (early stage group) or seven days (for the advanced stage group) after the lymphoma inoculation, and after the PET acquisition, the entire gastrointestinal tract (between the subphrenic segment of oesophagus and the upper third of rectum, together with the adjoining mesentery) was removed and placed in a 10 cm Petri dish, with the intestinal folds flattened. Under gentle pressure, the dissected gut-mesentery complex was fixed in cold 4% buffered paraformaldehyde for 15 min, followed by repeated rinsing with PBS.

### 4.6. Autoradiography

Phosphor imager plate autoradiography (ARG) was performed on the fixated gastrointestinal tract preparations 2 h post harvesting the organs. A GE Typhoon 9400 (General Electric, Boston, MA, USA) imager was used with the sections incubated for 10 min on the plates, with 100 micron voxel size. The dedicated image export function was applied to obtain .tiff format high-resolution images that were in turn visualized with either ImageJ or VivoQuant software.

### 4.7. Cerenkov Light Imaging Ex Vivo

Following the end of PET acquisition, Cerenkov light imaging using Cerenkov luminescence (produced in the water of tissues with high concentration of the injected radionuclidic tracer) was performed using a prototype high resolution Cerenkov imaging system with an exceptionally sensitive lens (LighPath^TM^, Lightpoint Medical, Chesham, UK). Tissue specimens were placed in the sample holder of the instrument and image acquisition was performed over them for 10 min of signal integration, with 4 × 4 pixel binning in 512 × 512 pixels, with a 0.1 mm pixel size. A black-and-white light photographic background was also collected by the instrument for anatomical reference. Images of Cerenkov luminescence and the background white-light images were exported to DICOM format by the instrument’s dedicated software. Further two-dimensional rigid co-registration, post-processing with a median filter of 3 pixels kernel, rotation, and color look-up table changes were performed using Fusion (Mediso, Budapest, Hungary) and VivoQuant (inviCRO, Boston, MA, USA). Images were then visualized with a unified common colour Look-Up Table (LUT) across animals, with arbitrary units of light intensity based on the emCCD detector pixel counts overlaid on the black-and-white photographs.

### 4.8. Fiber-Optic Confocal Endomicroscopy Imaging of FITC Molecules Ex Vivo

The S1500 fiberoptic microscope probe tip (resolution 3.3 microns, field-of-view diameter, 600 microns) of the Cellvizio Lab Dual Band imaging system (Mauna Kea Technologies Inc., Paris, France) was applied to the lymph nodes and 480 nm green bandwidth live microscopic images of the intestinal walls, peritoneal surfaces, and lymph nodes or visible lymphatic vessels were collected. Using this method, individual tumor cells can be visualized by their FITC fluorescent dye content in the ex vivo intestinal organ preparations. The imaging was performed immediately after autopsy of the animals.

### 4.9. Immunohistochemistry

The CFSE-labeled lymphoma cells were detected using anti-FITC immunohistochemistry. First the gut-mesentery complex previously fixed in 4% buffered paraformaldehyde for 30 min was extensively washed in PBS, and was then incubated with 1 mg/mL phenyl-hydrazine hydrochloride (Sigma-Aldrich) in PBS to block endogenous peroxidase activity, followed by saturation in 5% BSA-PBS-0.1% saponin for 2 h at 4 °C with continuous shaking. HRP-conjugated sheep anti-fluorescein antibody (Southern Biotech) diluted in 0.1% BSA-PBS-0.1% saponin was added and incubated overnight with continuous shaking. After extensive washing in PBS-0.1% saponin, the immune reaction was visualized using DAKO diamino-benzidine/H_2_O_2_ substrate in 5 mM TRIS buffer at pH 7.2.

The immunohistochemical staining for Ki-67 and B220 antigens was performed as previously described [11].

Lymphoma-infiltrated mesentery and mLNs were embedded in Killik freezing medium and were frozen. Frozen sections with a thickness of 8 µm were cut using a Leica cryostat and were allowed to dry overnight. After fixing in cold acetone for 5 min, the sections were dried and rehydrated in 1 mg/mL phenyl-hydrazine-PBS solution for 20 min. Slides were incubated with anti-Ki-67 rat mAb (clone #11F6 from Biolegend (Biomedica Hungária Kft), Budapest, Hungary, at 1:100 dilution in PBS) or anti-B220 hybridoma supernatant (clone #RA3-6B2) for 45 min, followed by washing. Next, the sections were incubated with ImmPRESS goat anti-rat IgG-HRP polymeric conjugate (Vector Laboratories, BioMarker, Gödöllő, Hungary) for 45 min, and after washing the sections were visualized using DAKO diamino-benzidine/H_2_O_2_ substrate in 5 mM TRIS buffer with pH 7.2.

## 5. Conclusions

Our clinically translatable findings point to the early detection possibilities and non-random metastatic route preferences of this lymphoma model. Local FDG administration should be given more consideration in radioisotopic diagnostic procedures. The adequate assessment of early peritoneal metastases in lymphomae, and perhaps clinically more importantly in colon, stomach or ovarian cancers, might be achieved this way. Topical diagnostic applications and the combination of imaging modalities are a clinically explorable method to promote more effective personalized topical therapy, in addition to systemic treatments. Cerenkov imaging translated to the clinic is one such avenue to achieve this.

## Figures and Tables

**Table 1:** Mouse lymphoma cell and healthy mixed mouse leukocyte [18F]FDG uptake differences. Bc.DLFL1 lymphoma cells have a significantly higher capacity to take up FDG over normal mouse leukocytes.

BcDLFL.1 Mouse Lymphoma Cells (n = 6 Measurements)		Healthy Mouse White Blood Cells (n = 6 Measurements)	Mean Lymphoma to Healthy Cell Uptake Proportions
Mean (SD) Ratio of Counts Per Minute		Mean (SD) Ratio of Counts Per Minute	14.77
Cell Pellet to Whole Incubate	47.56% (3.22%)	2.77% (2.22%)	

## Data Availability

The data that support the findings of this study are available from the corresponding author upon reasonable request.
